# Advancing the Paradigm of Temporal Lobe Epilepsy as a Network Disease: The Promise of Biomarkers and Targeted Disease Modification

**DOI:** 10.3390/ijms27188328

**Published:** 2026-09-19

**Authors:** Károly Orbán-Kis, Krisztina Kelemen, Rita-Judit Kiss, Zsolt Gáll, Zsolt András Nagy, Anna Fehér, Nándor Todor, Ádám Szentes, Júlia Erzsébet Metz, Tibor Szilágyi

**Affiliations:** 1 Department of Physiology, George Emil Palade University of Medicine, Pharmacy, Science, and Technology of Târgu Mureș, 540142 Târgu Mureș, Romania; karoly.orban-kis@umfst.ro (K.O.-K.); rita.kiss@umfst.ro (R.-J.K.); julia.metz@umfst.ro (J.E.M.); tibor.szilagyi@umfst.ro (T.S.); 2 Department of Pharmacology and Clinical Pharmacy, George Emil Palade University of Medicine, Pharmacy, Science and Technology of Târgu Mureș, 540142 Târgu Mures, Romania; zsolt.gall@umfst.ro; 3 Faculty of Medicine, George Emil Palade University of Medicine, Pharmacy, Science, and Technology of Târgu Mureș, 540142 Târgu Mureș, Romania; zsolt.andras.nagy@gmail.com (Z.A.N.); annafeher03@gmail.com (A.F.); nandort88@gmail.com (N.T.); szentesadam1234@gmail.com (Á.S.)

**Keywords:** temporal lobe epilepsy, epileptogenesis, neuroinflammation, disease-modifying therapies, biomarkers, precision medicine, antiseizure medications

## Abstract

Temporal lobe epilepsy (TLE) is increasingly conceptualized not as an isolated hippocampal lesion, but as a complex, multiscale limbic network connectomic disorder. Despite advances in pharmacological management, over 30% of patients experience drug-resistant epilepsy, underscoring the urgent need to shift from symptomatic seizure control to mechanism-directed disease modification. This review comprehensively synthesizes the pathophysiological architecture of epileptogenesis in TLE, spanning mitochondrial bioenergetic alterations, chronic neuroinflammation, synaptic reorganization, and ionic plasticity resulting from ion-channel dysregulation. We evaluate diagnostic advancements, highlighting how invasive stereo-EEG disambiguates pathological high-frequency oscillations from physiological ripples, how structural HARNESS-MRI maps anatomical substrates, and how AI-driven algorithms analyze ultra-long-term EEG streams for continuous seizure forecasting. Additionally, peripheral biofluid proteins and microRNAs provide noninvasive windows into active neuroinflammation and network remodeling, serving as valuable tools for longitudinal disease monitoring rather than primary screening. Therapeutically, the field is evolving beyond empirical antiseizure medications toward mechanism-based rational drug design and precision interventions. Dual-mechanism agents enhance seizure freedom, while antisense oligonucleotides, microRNA antagomirs, and cation-chloride cotransporter modulators target underlying genetic and biophysical drivers. Minimally invasive ablation, AI-guided closed-loop neuromodulation, targeted anti-inflammatory biologics, and patient-derived 3D cerebral organoid platforms further expand the translational frontier. Ultimately, bridging these experimental modalities through prospective clinical validation may provide a viable path toward interrupting epileptogenesis and realizing true disease modification in human TLE.

## 1. Introduction

Epilepsy is a common, chronic neurological disorder affecting over 50 million people globally [1,2]. It is characterized by an enduring predisposition to generate unprovoked seizures, heavily burdened by substantial psychosocial and cognitive consequences [2]. While modern neuropharmacology has introduced an extensive array of antiseizure medications (ASMs), the clinical reality still is that at least 30% of patients fail to achieve seizure freedom, a formidable rate of pharmacoresistance that is particularly pronounced in temporal lobe epilepsy (TLE) [3,4,5].

A fundamental distinction in modern epileptology separates ictogenesis, the immediate generation of a seizure, from epileptogenesis [6,7,8]. Epileptogenesis is the latent, multiphasic process in which structurally or functionally injured brain tissue undergoes cascading molecular, cellular, and network-level alterations, ultimately establishing a hyper-excitable state [6,7,9]. Modern translational neuroscience is driving a paradigm shift in how epilepsy is conceptualized. Epilepsy is increasingly viewed as a multiscale network connectomic disorder involving structural nodes, macro-scale hubs, altered glial networks, and neurovascular unit adaptations [10,11,12]. Recent investigations suggest that alterations in genomic integrity, mitochondrial energetic dysfunction, and chronic neuroinflammation may also contribute to the pathophysiology of TLE [13,14,15]. Reversing or halting this process is the paramount goal of modern translational epileptology, necessitating a pivot from merely suppressing symptoms to deploying disease-modifying therapies [5,6,7].

## 2. Classification of Epilepsy and Epileptic Seizures

To optimize clinical applicability, the International League Against Epilepsy updated its framework in the 2025 operational classification, which categorizes epilepsy by seizure type (focal, generalized, or unknown), epilepsy type, and underlying etiology [16]. The new classification emphasizes the distinction between focal and generalized seizures and incorporates awareness as a key descriptor of focal seizure semiology. Although validated in secondary care settings [17], this system remains clinically descriptive rather than mechanics-based, as underlying pathophysiological drivers often remain elusive. Notably, seizures are merely the overt clinical manifestations of diverse epileptogenic processes encompassing structural, genetic, infectious, metabolic, immune, or, quite often, cryptogenic factors. Consequently, seizures represent common symptomatic manifestations of distinct underlying pathologies, a heterogeneity that precludes a one-size-fits-all therapeutic solution [18].

TLE frequently falls under the structural umbrella, predominantly manifesting alongside mesial temporal sclerosis [19]. Nevertheless, modern genomics has also uncovered critical ion channel mutations (e.g., SCN1A, KCNQ2) and epigenetic regulators that demand clinicians to evaluate genetic and structural substrates simultaneously, shifting from descriptive phenomenology toward etiology-driven precision medicine [18,20].

## 3. Temporal Lobe Epilepsy: Pathomechanisms of Epileptogenesis

### 3.1. The Role of the Hippocampus and Network Connectomics

The hippocampus has long been regarded as a key structural hub of TLE owing to its laminated tri-synaptic architecture being intrinsically vulnerable to hypersynchronization and seizure-induced collapse [12,21]. A central component of mesial temporal epileptogenesis is failure of the dentate gyrus gate. Loss of hilar mossy cells and interneurons, altered granule-cell intrinsic excitability, and aberrant recurrent excitation collectively reduce the ability of the dentate gyrus to restrict entorhinal excitation from entering the hippocampal circuit [22,23].

However, a growing body of network connectomics evidence reframes TLE as a multinodal limbic-network disorder. The hippocampus does not operate in isolation. Rather, it functions as one node within an integrated, highly interconnected network of limbic and subcortical structures that are co-involved in TLE pathobiology [10,24,25]. Structures including the basolateral amygdala, piriform cortex, entorhinal and perirhinal cortices, endopiriform nucleus, and thalamic relay nodes such as the paraventricular nucleus of the thalamus exhibit profound pathological involvement and may contribute to network hyperexcitability and seizure propagation in some patients [26,27]. Seizure-induced metabolic overload across these interconnected nodes can promote compounding mitochondrial dysfunction, oxidative stress, and cumulative DNA damage [13,28]. Connectomic analyses demonstrate that the emergence of epileptic seizures may involve high-connectivity “rich-club” hubs distributed throughout this mesial temporal and subcortical circuit [29,30,31]. Reciprocal excitatory feedback and compromised local surround inhibition across these hubs facilitate hypersynchrony, ultimately driving progressive structural and functional alterations in distant neocortical networks [30,32,33].

### 3.2. Cell Loss, Reorganization, and Ionic Plasticity

Hippocampal sclerosis involves the loss of CA1 and CA4 pyramidal cells [34]. Proteomic profiling reveals that within the sclerotic hippocampus, proteins involved in complement system activation, astrocyte reactivity, and extracellular matrix organization are upregulated, whereas synaptic proteins are downregulated [35,36]. In addition to principal-cell loss, selective vulnerability of hilar mossy cells and abnormal survival of excitatory mossy-cell subpopulations contribute to dentate network disinhibition and recurrent excitation [22,37]. Synaptic reorganization, frequently observed in human TLE tissue and animal models, is characterized by mossy fiber sprouting in the dentate gyrus alongside severe ionic plasticity at GABAergic synapses [38,39]. Concomitant granule-cell dispersion and the appearance of ectopic granule cells with aberrant basal dendrites further destabilize dentate microcircuitry and promote recurrent excitation [40].

The pathology-dependent collapse of transmembrane ion gradients, most notably chloride homeostasis, fundamentally alters how neurons respond to inhibitory neurotransmitters [38,41]. Neuroinflammatory and neurotrophic signaling cascades (e.g., BDNF/TrkB) may contribute to altered chloride homeostasis by reducing KCC2-mediated chloride extrusion and increasing NKCC1-mediated chloride uptake [42,43]. The process can create a pathological state of intracellular chloride accumulation. The chloride equilibrium potential is consequently shifted to a depolarized state, above the resting membrane potential. Under these conditions, synaptic GABA release and GABA_A_ receptor activation can become depolarizing under certain conditions, reducing the efficacy of GABAergic inhibition [42,44]. However, this shift is not uniformly present in epileptogenesis and is not specific to epileptic processes. Nevertheless, when present, the severe ionic shift may functionally collapse the surround inhibition necessary to contain focal discharges, permitting seizures to propagate unimpeded across the limbic network, and may contribute to reduced responsiveness to some GABA-enhancing antiseizure medications in specific contexts [33,41,45].

### 3.3. Voltage-Gated and Mitochondrial Ion Channels

At the single-cell level, paroxysmal depolarization shifts (PDS) may accompany ictogenesis in susceptible neuronal populations. These events comprise a prolonged, high-amplitude depolarizing membrane potential envelope accompanied by high-frequency bursts of action potentials [46,47]. The initiating voltage envelope of the PDS is driven by the massive influx of sodium and calcium ions through the voltage-gated ion channels [46,48]. Crucially, pathological amplifications of the persistent, non-inactivating sodium current (mediated by altered voltage-gated sodium channel subunit kinetics, e.g., Na_v_1.1, Na_v_1.6 dysregulation) lower the threshold for action potential generation and sustain subthreshold membrane potential oscillations [47,49]. Concurrently, activation of low-voltage-activated (T-type) and high-voltage-activated (L-type, N-type, P/Q-type) calcium channels sustains the prolonged plateau depolarization [48,50]. This concerted channelopathy may transform physiological principal neurons into autonomous, intrinsic bursting units capable of pacing and orchestrating hypersynchronous network firing across local microcircuits [50,51].

Beyond plasmalemmal ion channels, mitochondrial ion channels and energetic cascades may contribute to neuronal survival and chronic network stability during repetitive seizure activity [52,53]. The massive cytosolic calcium surges accompanying the PDS force the mitochondrial calcium uniporter into overdrive, pulling excessive amounts of calcium into the mitochondrial matrix to buffer the cytosolic load [13,54]. However, in many experimental models of recurrent seizures, chronic intra-mitochondrial Ca^2+^ overload, compounded by elevated reactive oxygen species generated during metabolic hyperactivity, triggers the pathological opening of the high-conductance mitochondrial permeability transition pore [13,55]. This in turn leads to the rapid dissipation of the mitochondrial membrane potential, uncoupling of oxidative phosphorylation, and severe ATP depletion [13,53]. This energetic failure impairs energy-dependent ion pumps (e.g., Na^+^/K^+^-ATPase), preventing membrane repolarization and establishing a self-sustaining vicious cycle where metabolic collapse fuels persistent electrical hyperexcitability and apoptotic cell death [52,53]. Consequently, targeting mitochondrial ion channels and metabolic regulators represents a critical strategy for restoring cellular bioenergetics and achieving neuroprotection [52,54].

### 3.4. Spatiotemporal Dynamics and Neurotransmitter Systems

Seizure generation and propagation represent a complex spatiotemporal process defined by precise chronological sequences across time and multiscale anatomical recruitment across space. Temporally, ictogenesis does not always start as an immediate excitatory outburst; rather, it is paradoxically preceded in the pre-ictal phase by escalating, highly synchronized firing of specific inhibitory interneuron subpopulations [56,57,58]. Fast-spiking parvalbumin-positive basket cells (providing perisomatic inhibition [59]) as well as neurons targeting distal dendrites (such as somatostatin-expressing interneurons [60] and Ivy cells [61]) exhibit pre-ictal hyperactivation [56]. Spatially confined at first within local microcircuits, this intense interneuron activity drives localized extracellular potassium accumulation and intracellular chloride loading (compounding ionic plasticity) [57,58,62]. Extracellular matrix remodeling, including degradation of perineuronal nets around parvalbumin interneurons, further destabilizes fast-spiking inhibition and constrains pathological plasticity in the epileptic temporal lobe [63]. Although interneurons can initially provide the rhythmic inhibitory timing that organizes principal-cell activity, subsequent impairment of inhibitory control—through depolarization block, altered chloride homeostasis, or activity-dependent failure—may allow recurrent excitation and intrinsic cellular mechanisms to promote the synchronized recruitment of pyramidal neurons during ictal onset [57,58].

In parallel, impaired adenosine signaling constitutes a key failure of endogenous seizure control. Upregulation of astrocytic adenosine kinase and altered A_1_/A_2_A receptor expression reduce extracellular adenosine tone and weaken presynaptic inhibition of glutamate release in sclerotic hippocampus [64,65].

Once initiated, the spatial dynamics shift from local microcircuit entrainment to macro-scale network recruitment, probably driven primarily by hyperactivation of the glutamatergic system. NMDA and AMPA receptor activation overcomes local surround inhibition, serving as the metabolic and electrophysiological engine for spatial seizure propagation across interconnected limbic hubs and distant neocortical structures, simultaneously mediating excitotoxic neuronal injury [33,66]. Beyond glutamate and GABA, neuromodulatory systems such as neuropeptide Y, endogenous opioids, and monoamines shape seizure threshold and propagation in the temporal lobe. Seizure-induced upregulation of neuropeptide Y and activation of presynaptic Y_2_ receptors provide an endogenous brake on glutamatergic transmission that may be insufficient to prevent ictogenesis in chronic TLE [67,68].

Intracellularly, these spatiotemporal dynamics seem to be structurally anchored by the mechanistic target of rapamycin signaling pathway. Pathological hyperactivation of this cascade contributes to structural epileptogenesis in specific genetic and acquired contexts by altering the spatial geometry of neural circuits, driving aberrant protein synthesis, dysregulated dendritic arborization, and abnormal mossy fiber sprouting, thereby creating a permanent, highly susceptible micro-architectural substrate for recurrent ictogenesis [69,70].

### 3.5. Neuroinflammation, Glial Reactivity, and Calcium-Binding Protein Remodeling

In several acquired epilepsies, chronic neuroinflammatory signaling is increasingly recognized as an important contributor to epileptogenesis rather than merely a secondary response to seizures [14,71]. Following tissue injury or cellular stress, endogenous damage-associated molecular patterns (DAMPs), notably High Mobility Group Box 1 (HMGB1), may be released and can activate innate immune pathways such as Toll-like receptor 4 (TLR4) signaling in microglia and astrocytes [14,72,73]. This activates the NOD-like receptor protein 3 (NLRP3) inflammasome and fuels the persistent secretion of pro-inflammatory cytokines, including IL-1β and TNF-α [14,74,75]. Elevated IL-1β lowers seizure thresholds by modulating excitatory and inhibitory transmission (e.g., by phosphorylating NMDA receptor subunits and downregulating ionotropic GABA_A_ receptors) [76,77]. Simultaneously, persistent microglial expansion and reactive astrogliosis spread across the entire limbic network [71,78]. Over extended periods, matrix metalloproteinases (e.g., MMP-9) degrade blood–brain barrier tight junctions, permitting albumin extravasation to engage astrocytic TGF-β receptors [79,80,81]. This cascade downregulates EAAT1/EAAT2 glutamate transporters and redistributes aquaporin-4 (AQP4) water channels, driving interstitial edema and chronic network hyperexcitability [79,80,82]. Astrocytic dysfunction extends beyond inflammatory signaling to impaired homeostatic control of extracellular potassium, glutamate, and water. Downregulation of Kir4.1 channels and GLT-1/EAAT2 transporters, together with altered AQP4 polarization, compromises ion and neurotransmitter clearance and facilitates seizure initiation and propagation [83,84].

Beyond cytokine signaling, neuroinflammatory stress and persistent seizure activity fundamentally disrupt intracellular calcium homeostasis and inhibitory interneuron subpopulation diversity [85,86]. While classical EF-hand calcium-binding proteins (CaBPs) like parvalbumin (PV) and calretinin (CR) display region-specific excitotoxic vulnerability [85,86,87], recent preclinical findings suggest that the N-terminal EF-hand protein NECAB1, which is highly expressed in CB1/CCK-positive GABAergic interneurons, undergoes dynamic, region-dependent upregulation during chronic epileptogenesis [27]. In selected critical subcortical hubs, the observed increase in NECAB1 expression may represent an endogenous compensatory calcium-buffering mechanism designed to counteract excessive calcium influx and modulate thalamocortical excitability. However, this interpretation remains provisional because it is based on limited experimental evidence and requires independent replication across additional epilepsy models, brain regions, disease stages, and species. Conversely, the reported reduction of specific subpopulations co-expressing NECAB1 and CR in some epileptic circuit structures may indicate that inflammatory microenvironments may selectively impair specific inhibitory interneuron profiles required for network stability [27].

Interestingly, novel preclinical data regarding pharmacological interventions reveal a striking divergence in how antiseizure medications modulate this neuroinflammatory and glial architecture [67,88]. For example, ligands targeting synaptic vesicle glycoprotein 2A (SV2A), which interacts with synaptotagmin-1 to regulate calcium-dependent exocytosis and synaptic vesicle recycling [89], exhibit distinct immunomodulatory profiles. In the reported experimental model, first-generation SV2A ligands like levetiracetam effectively suppressed microglial activation, attenuated GFAP+ astrogliosis, and partially normalized NECAB1/CR expression across limbic nodes [90], whereas its higher-affinity analogue brivaracetam [91] unexpectedly markedly increased microglial density across circuit hubs [92]. This contrast suggests that microglial SV2A binding or differential off-target pathways may dynamically modulate microglial proliferation and neuroinflammatory tone independently of seizure control, highlighting the necessity of evaluating glial- and CaBP-targeted network pharmacology in antiepileptogenic drug design [67,92]. Nevertheless, this apparent contrast needs further validation before glial or calcium-binding-protein signatures can be considered reliable pharmacodynamic biomarkers or therapeutic targets in antiepileptogenic drug development.

## 4. Cognitive and Psychiatric Comorbidities in Epilepsy

The burden of TLE extends beyond seizures; it is strongly linked to progressive memory impairment, depression, and anxiety [93,94]. The classical view that these comorbidities are solely psychosocial reactions has been superseded by symptom-centered network pharmacology, revealing shared underlying biological networks [94,95]. Structural atrophy, inflammatory cytokine release, and extracellular matrix disruption actively dismantle cognitive networks [14,71]. Patients with higher systemic concentrations of HMGB1 and S100B tend to exhibit statistically significantly lower Montreal Cognitive Assessment scores [96,97]. Prolonged RAGE (Receptor for Advanced Glycation End-products) activation by these proteins induces mitochondrial malfunction and apoptosis in the hippocampus, contributing to distributed cognitive deficits [72,98]. Furthermore, the intersection of tau pathology and altered brain energetic metabolism may link premorbid psychiatric vulnerability to epileptogenesis [99]. Additionally, observational neuropsychiatric studies have reported that interictal personality disorders and pathological personality traits may be more prevalent in TLE cohorts than in controls. These findings indicate an association but do not establish causality, and the underlying contributions of limbic network alterations, neurodevelopmental vulnerabilities, neuroinflammatory stress cascades, psychosocial variables, and medication effects remain uncertain [100].

## 5. Diagnosing Epilepsy: Advanced Diagnostics and Biomarkers

### 5.1. EEG, sEEG, and High-Frequency Oscillations (HFOs)

While standard scalp EEG and long-term video-EEG remain the gold standards for confirming clinical diagnoses (Table 1), conventional scalp recordings suffer from severe signal attenuation, muscle artifacts, and spatial smoothing and their conventional bandwidths are insufficient for capturing micro-network pathologies [101]. Consequently, scalp EEG is generally insufficient for routine reliable detection of HFOs—subdivided into ripples (80–250 Hz) and fast ripples (250–500 Hz)—rendering these sensitive and rather specific electrophysiological phenomena unsuitable as noninvasive screening biomarkers [102,103]. Instead, capturing these micro-network pathologies requires invasive intracranial monitoring, particularly high-density stereoelectroencephalography (sEEG) [101]. Crucially, HFOs also exert important physiological functions; physiological ripples generated in the hippocampus and neocortex facilitate memory consolidation, synaptic plasticity, and information transfer during quiet wakefulness and slow-wave sleep [104]. In contrast, pathological ripples and fast ripples reflect the population bursts of hypersynchronous epileptogenic networks [105,106]. Disambiguating, during invasive sEEG recordings, the physiological HFOs from their pathological counterparts and tracking their transition into abnormal synchronized events provides actionable targets to map the epileptogenic zone and guide precision surgical or neuromodulatory interventions [103,107].

### 5.2. Structural Neuroimaging and Artificial Intelligence

Neuroimaging has experienced exponential growth, with the Harmonized Neuroimaging of Epilepsy Structural Sequences (HARNESS-MRI) protocol establishing a standardized, high-field framework to detect subtle structural substrates like mesial temporal sclerosis (MTS) and focal cortical dysplasia [108]. Similarly, functional MRI (fMRI) maps distal network disruptions, such as altered temporal-prefrontal connectivity, useful in drug-resistant patients [10]. However, with the notable exception of congenital malformations of cortical development such as focal cortical dysplasia, these macroscopic structural and functional alterations typically manifest late in the progressive epileptogenic cascade [7,9]. Consequently, while neuroimaging aids advanced treatment planning, it is unlikely to enable early prevention of epileptogenesis on its own [9]. Furthermore, image-guided surgical resections or network disconnections do not restore the brain’s physiological baseline; rather, they provide symptomatic relief by halting seizure propagation [9,109].

Concurrently, artificial intelligence (AI) is driving a proactive paradigm shift in seizure forecasting by processing ultra-long-term EEG streams, sometimes combined with continuous multimodal ambulatory data with heart-rate variability and accelerometry [110,111,112,113,114,115,116]. Although predicting seizures in advance may allow for the timely initiation of rescue treatments, this AI-driven approach remains a compensatory management strategy that neither arrests the underlying epileptogenic process nor provides a definitive cure for the patient [9,71,116].

### 5.3. Fluid Biomarkers

In peripheral biofluids, primarily blood, cerebrospinal fluid and emerging matrices such as saliva and biofluid-derived extracellular vesicles (exosomes), elevated concentrations of HMGB1 (a prototypical DAMP) and the astroglial protein S100B have emerged as candidate biomarkers of active neuroinflammation as well as associated cognitive decline [117]. Since these markers are influenced by multiple comorbidities (e.g., trauma, systemic inflammation, neurodegeneration), current data indicate low positive predictive value in unselected populations. Additional fluid biomarkers including nesfatin-1, total and phosphorylated tau variants, and amyloid-beta (Aβ42) are actively investigated to dissect the bidirectional link between epileptogenesis and neurodegeneration [99]. Furthermore, circulating and exosomal microRNAs (miRNAs) are heavily dysregulated in TLE, offering a noninvasive molecular window into active tissue pathology [118,119]. Beyond miRNAs implicated in accelerated biological aging (e.g., miR-34a, miR-146a), critical non-aging-related microRNAs directly orchestrate network hyperexcitability and structural reorganization [119]. Notably, brain-enriched miR-134 regulates dendritic spine morphogenesis and synaptic plasticity; its upregulation directly enhances network excitability, whereas anti-miR-134 silencing attenuates seizure susceptibility [120]. Similarly, activity-dependent miRs such as miR-132 (governing activity-regulated synaptogenesis and mossy fiber sprouting) and miR-124 (modulating neuronal excitability and microglial phenotypes) are significantly altered in TLE, highlighting a broader epigenetic architecture driving epileptogenesis independently of aging cascades [119].

Despite their clinical potential, significant methodological and biological limitations restrict the deployment of fluid biomarkers [121]. A major hurdle is their limited standalone sensitivity and specificity: peripheral trauma, systemic autoimmune or inflammatory conditions, and co-occurring neurodegenerative disorders frequently alter biofluid protein levels, creating substantial diagnostic noise [117,121]. Consequently, fluid biomarkers exhibit low positive predictive value in unselected populations, rendering them unsuitable as primary screening tools. Instead, their true diagnostic strength lies in longitudinal disease monitoring such as tracking active neuroinflammation, blood–brain barrier dysfunction, and disease trajectory post-insult (e.g., after status epilepticus or traumatic brain injury) [67,121].

Beyond inflammatory and neurodegenerative markers, the adenosine system represents a mechanistically grounded biomarker and therapeutic target. Increased adenosine kinase expression and altered A1/A2A receptor profiles in resected TLE tissue correlate with impaired endogenous seizure control and may inform future gene-therapy approaches [64,65].

When appropriately integrated, specific biomarker profiles can directly inform precision therapeutics [67]. For instance, detecting persistent elevations of HMGB1 or pro-inflammatory cytokines may help identify patients who could be considered for targeted anti-inflammatory regimens, including IL-1R1 antagonism with anakinra, experimental inhibition of the HMGB1/TLR4 pathway, or NLRP3-targeted interventions, thereby moving fluid biomarker profiling beyond passive monitoring toward mechanism-informed therapeutic stratification [67,75,122]. However, this approach is largely investigational, and even these targeted immunomodulatory strategies would be non-curative. By dampening neuroinflammatory cascades and restoring microenvironmental stability these strategies elevate the seizure threshold and effectively suppress active ictogenesis, but they do not fully reverse established structural network remodeling or eradicate the underlying epileptogenic substrate [67,123].

**Table 1:** Diagnostic, localization, prognostic, and translational tools relevant to temporal lobe epilepsy (see text for references).

Investigation Method/Biomarker	Principal Information Obtained	Invasiveness	Current Clinical or Research Role	Key Limitation	Diagnostic and Translational Role
Routine scalp EEG	Interictal epileptiform discharges, background activity, focal slowing, and seizure patterns.	Non-invasive	Initial diagnostic evaluation and syndrome classification.	Limited sensitivity; a normal EEG does not exclude epilepsy; artifacts and poor spatial resolution.	Diagnosis and seizure characterization.
Long-term video-EEG monitoring	Electroclinical seizure correlation, seizure semiology, ictal onset, and differential diagnosis of paroxysmal events.	Non-invasive	Reference method for electroclinical characterization and presurgical assessment.	Time-consuming, resource-intensive, and vulnerable to scalp-recording limitations.	Diagnosis, seizure characterization, and presurgical evaluation.
Ambulatory EEG	Interictal and ictal activity during prolonged outpatient monitoring.	Non-invasive	Extended monitoring when inpatient video-EEG is unavailable or unnecessary.	Limited video/semiological information; variable signal quality and adherence.	Diagnosis and longitudinal seizure monitoring.
Stereo-EEG (sEEG)	Intracranial ictal onset, interictal discharges, HFOs, and spatial relationships within epileptic networks.	Invasive; intracranial	Presurgical localization and functional network mapping.	Requires implantation; risks include hemorrhage, infection, and sampling error.	Localization and characterization of epileptogenic networks
Structural MRI (HARNESS-MRI)	Mesial temporal sclerosis, focal cortical dysplasia, tumors, vascular lesions, and other structural substrates.	Non-invasive	Standard structural evaluation and surgical planning.	May be negative in MRI-negative epilepsy; subtle abnormalities can be missed; does not directly measure epileptogenicity.	Etiological classification and structural localization.
Quantitative MRI and volumetry	Hippocampal volume, cortical thickness, signal abnormalities, and asymmetry.	Non-invasive	Research, quantitative assessment, and selected presurgical applications.	Requires standardized acquisition and normative databases; limited individual-level specificity.	Structural characterization, prognosis, and research biomarker development.
Diffusion MRI/DTI	White-matter microstructural abnormalities and altered structural connectivity.	Non-invasive	Research and adjunctive network characterization.	Methodological variability and limited specificity for routine diagnosis.	Network characterization and prognosis.
Functional MRI	Altered resting-state connectivity, language and memory lateralization, and network-level dysfunction.	Non-invasive	Adjunctive presurgical assessment and research.	Motion and physiological artifacts; limited standardization and uncertain individual-level interpretation.	Network characterization, functional mapping, and presurgical planning.
FDG-PET	Interictal cerebral glucose hypometabolism and functional abnormalities	Minimally invasive	Localization in selected drug-resistant or MRI-negative cases.	Limited spatial specificity; hypometabolism may extend beyond the epileptogenic zone.	Functional localization and network characterization
Ictal/interictal SPECT	Perfusion changes associated with seizure onset and propagation.	Minimally invasive	Selected presurgical localization, particularly when other data are discordant.	Requires rapid tracer administration; expensive and technically demanding; limited temporal resolution.	Ictal localization and seizure-propagation analysis.
MEG	Interictal magnetic dipoles and network-level electrophysiological abnormalities.	Non-invasive	Adjunctive localization in selected presurgical cases.	Limited availability, cost, susceptibility to artifacts, and variable sensitivity.	Electrophysiological localization and network characterization.
Neuropsychological assessment	Memory, language, executive function, and lateralizing cognitive profiles.	Non-invasive	Baseline characterization, lateralization, surgical risk assessment, and outcome monitoring.	Influenced by education, mood, medication, seizure burden, and cognitive reserve.	Functional characterization, prognosis, and treatment planning.
Genetic testing	Pathogenic variants associated with monogenic epilepsies or genetic susceptibility.	Minimally invasive	Etiological diagnosis and precision-medicine assessment in selected patients.	Limited diagnostic yield in many adult-onset TLE cases; variants of uncertain significance; findings may not establish causality	Etiological classification and therapeutic stratification.
Serum and CSF autoimmune antibodies	Antibodies associated with autoimmune encephalitis and immune-mediated seizures.	Minimally invasive or invasive, depending on CSF sampling.	Etiological evaluation when autoimmune epilepsy is clinically suspected.	Interpretation requires clinical correlation; false positives and false negatives occur; not a general screening test for TLE.	Etiological classification and immune-mechanism identification.
Fluid inflammatory biomarkers	Candidate markers of neuroinflammation, blood–brain barrier dysfunction, or tissue injury, including HMGB1 and S100B.	Minimally invasive or invasive	Exploratory research and longitudinal monitoring.	Limited specificity and sensitivity; affected by systemic inflammation, trauma, age, and comorbidity.	Mechanistic profiling, prognosis, and longitudinal monitoring.
Tau and amyloid-related biomarkers	Molecular signatures potentially linking epilepsy with neurodegenerative processes.	Minimally invasive or invasive	Research and investigation of cognitive decline or neuro-degenerative overlap.	Not validated for routine epilepsy diagnosis; substantial overlap with other neurological disorders.	Neurodegenerative profiling and prognosis.
Circulating and exosomal microRNAs	Candidate signatures associated with synaptic remodeling, excitability, inflammation, and aging-related pathways.	Minimally invasive	Exploratory biomarker discovery and potential longitudinal monitoring.	Pre-analytical variability, lack of standardized assays, and limited independent validation.	Molecular profiling, prognosis, and therapeutic stratification research.
Adenosine-system markers	Adenosine kinase and A1/A2A receptor alterations associated with endogenous seizure control	Usually tissue-based; indirect assessment may be possible	Experimental biomarker and therapeutic-target research.	Limited accessibility in patients; not currently a routine clinical biomarker.	Mechanistic characterization and therapeutic-target identification.
AI-based EEG analysis	Automated seizure detection, interictal discharge recognition, and seizure forecasting.	Non-invasive or minimally invasive, depending on recording modality	Decision support and research; selected detection systems are entering clinical use.	Generalizability, dataset bias, false alarms, lack of external validation, and limited interpretability.	Diagnosis, seizure monitoring, forecasting, and adaptive-treatment research.
Wearable multimodal monitoring	Convulsive seizure detection and physiological changes involving movement, heart rate, or electrodermal activity.	Non-invasive	Home monitoring and investigational seizure forecasting.	Reduced sensitivity for focal aware and non-motor seizures; patient-specific performance varies.	Longitudinal monitoring, safety assessment, and seizure forecasting.
iPSC-derived neurons, organoids, and assembloids	Cellular excitability, network activity, genetic phenotypes, and drug responses.	Ex vivo research models	Mechanistic investigation and preclinical drug prioritization.	Immature or incomplete cellular organization; limited vascular, immune, and whole-brain representation; not routine clinical diagnostics.	Mechanistic modeling, target discovery, therapeutic screening, and translational validation.

## 6. Therapeutic Perspectives and Precision Epileptology

### 6.1. Rational Drug Design and Targeted RNA Therapeutics

Modern translational pharmacology is undergoing a fundamental shift from empirical screen-based drug discovery toward mechanism-based rational drug design; this approach is true for epilepsy as well [124,125,126]. High-resolution cryo-electron microscopy and advanced computational docking models now resolve atomic-scale conformational states of ion channels and synaptic receptors, enabling the structural engineering of small molecules that bind to highly specific allosteric pockets with minimized off-target liabilities. A hallmark of this structural revolution is the development of dual-mechanism agents designed to target complementary pathways of hyperexcitability simultaneously. A prime exemplar is cenobamate, which exerts dual action by selectively inhibiting the persistent, non-inactivating sodium current (thereby suppressing autonomous repetitive firing) while concurrently acting as a positive allosteric modulator of non-benzodiazepine binding sites on GABA_A_ receptors [124,127]. This synergistic polypharmacological profile restores network inhibition and yields high seizure reduction rates in refractory focal epilepsies, establishing a template for next-generation multi-target antiseizure drug discovery [127]. It must be noted, however, that, while such multi-target small molecules achieve vastly superior seizure control, they remain fundamentally antiseizure (symptomatic) rather than antiepileptogenic (disease-modifying); they effectively suppress active ictogenesis without reversing the underlying progressive structural, glial, or connectomic network remodeling [126].

Parallel to small-molecule rational design, targeted RNA therapeutics, most notably antisense oligonucleotides (ASOs) and microRNA antagomirs, offer a radical mechanism to modulate traditionally non-druggable genetic and epigenetic targets underlying epileptogenesis [120,128]. ASOs leverage sequence-specific base pairing to degrade targeted RNA transcripts via RNase H recruitment, alter pre-mRNA splicing, or augment protein translation from healthy alleles [128,129]. In developmental and epileptic encephalopathies as well as selected genetic epilepsies, specific ASO therapies have shown substantial preclinical promise, with some modalities progressing into early clinical development. These approaches provide important proof-of-principle examples of how genetically defined epilepsies may be treated by correcting or compensating for specific molecular defects. A prototypical representative example is STK-001 (zorevunersen), an ASO utilizing Targeted Augmentation of Nuclear Gene Output (TANGO) technology to selectively alter alternative splicing and upregulate wild-type SCN1A transcript expression in Dravet syndrome, increasing Na_v_1.1 sodium channel expression levels in fast-spiking inhibitory interneurons [128]. Similarly, allele-specific knockdown ASOs are actively investigated to degrade mutant mRNA transcripts in gain-of-function SCN2A and KCNQ2 channelopathies, while ASOs targeting the UBE3A antisense transcript (UBE3A-ATS) unsilence paternal UBE3A expression in Angelman syndrome [129,130,131]. Beyond monogenic channelopathies, non-coding RNA-targeted ASOs, such as anti-miR-134 antagomirs, selectively silence pathogenic microRNA-134 to prevent aberrant dendritic spine morphogenesis, attenuate synaptic remodeling, and show antiepileptogenic effects in experimental TLE models [120]. Importantly, these genetically targeted approaches are presented here as illustrative examples of mechanism-based precision therapy and should not be interpreted as established or currently applicable treatments for temporal lobe epilepsy, which is etiologically and molecularly more heterogeneous.

In acquired or non-monogenic TLE, actionable targets are unlikely to consist of recurrent pathogenic variants analogous to those found in developmental genetic epilepsies [132]. Instead, target discovery will probably require molecular stratification based on convergent abnormalities in neuroinflammatory signaling, glial ion and glutamate homeostasis, synaptic plasticity, and network remodeling [132,133]. Integrated transcriptomic, epigenomic, and cell-type-specific analyses of epileptogenic hippocampal tissue may help distinguish causal mechanisms from secondary changes caused by recurrent seizures, medication exposure, or gliosis [134]. Dysregulated microRNAs and other non-coding RNAs could provide therapeutically relevant pathway-level targets, but their clinical translation will require functional validation, replication in independent cohorts, and evidence of disease modification rather than seizure suppression alone [132]. Consequently, RNA-based precision therapy for acquired TLE should currently be regarded as a stratified, investigational approach rather than a broadly applicable treatment [132,135].

### 6.2. Targeting Ionic Plasticity and Chloride Homeostasis

Given the critical role of pathological intracellular chloride accumulation in driving refractoriness to classical GABAergic antiseizure medications, targeting ionic plasticity has emerged as a major frontier in precision epileptology [6,136,137]. Standard GABA_A_ receptor PAMs (e.g., benzodiazepines, barbiturates) rely on a physiological hyperpolarizing chloride influx [38]. When epileptogenic KCC2 downregulation and NKCC1 upregulation collapse the transmembrane chloride gradient, enhancing GABA_A_ channel opening paradoxically induces depolarizing chloride efflux, which may help explain reduced efficacy of some GABAergic drugs in subsets of patients and experimental models [38,136]. Rather than merely modulating channel gating kinetics [138], next-generation therapeutic strategies aim to restore the fundamental driving force of inhibitory currents by re-establishing the physiological intracellular chloride baseline [6,137,139].

This strategy is actively pursued through two complementary pharmacological avenues: suppressing pathological chloride influx via selective NKCC1 cotransporter inhibitors and accelerating chloride extrusion via KCC2 positive allosteric modulators [137,139]. While the prototypical NKCC1 blocker bumetanide established initial proof-of-concept, its poor blood–brain barrier permeability and potent systemic diuretic effects limited its clinical utility [137,140]. To overcome these pharmacokinetic constraints, lipophilic, brain-penetrant prodrugs and targeted bumetanide analogues (such as STS-66) have been engineered for enhanced CNS bioavailability [140,141]. Concurrently, small-molecule KCC2 PAMs (such as CLP290 derivatives) act directly to enhance KCC2 cell-surface expression and transport kinetics without inducing systemic diuretic liabilities [139,142]. By resetting the chloride reversal potential well below the resting membrane potential, these agents may restore functional GABA-mediated hyperpolarization, rescue perilesional surround inhibition, and reverse pharmacoresistance directly at its biophysical root [136,139,142].

The therapeutic window for KCC2 enhancement may be developmentally and regionally constrained, because KCC2 expression, phosphorylation, and transport capacity differ among brain regions and neuronal subtypes; therefore, indiscriminate activation could alter physiological chloride-dependent signaling beyond the epileptogenic network [143,144,145]. Moreover, clinical translation of NKCC1 inhibition has been inconsistent: bumetanide did not demonstrate clear efficacy in the NEMO phase 1/2 trial of acute neonatal seizures and raised ototoxicity concerns [146,147], whereas evidence for adult acute seizures remains predominantly preclinical [148], emphasizing the importance of CNS exposure, seizure etiology, treatment timing, and biomarker-guided patient selection. Also, clinicians and translational researchers must exercise significant caution: all of these chloride-restoring strategies remain largely experimental and are primarily validated in preclinical rodent models and early-stage translational pipelines [6,137]. Transitioning these experimental molecules into clinical practice requires rigorous human trial validation to establish their long-term safety, CNS selectivity, and clinical efficacy before they can be safely integrated into human antiepileptogenic regimens [6,137,142].

### 6.3. Neuromodulation and Minimally Invasive Surgery

Advanced closed-loop neuromodulation strategies, including responsive neurostimulation, deep brain stimulation of thalamic nuclei, and vagus nerve stimulation, are shifting therapeutic paradigms from static, open-loop stimulation toward real-time, adaptive control [149,150]. Increasingly, AI and deep-learning predictive models are increasingly being integrated into these device architectures to continuously analyze intracranial electrocorticography and sEEG streams alongside ambulatory biosensors [110,111,114,151]. By detecting subtle pre-ictal network phase shifts rather than relying solely on post-onset thresholding, AI-driven reactive neuromodulation enables proactive, personalized electrostimulation to disrupt hypersynchronous population firing seconds to minutes before clinical manifestations occur [111,150].

At the surgical frontier, minimally invasive innovations such as Magnetic Resonance-guided Laser Interstitial Thermal Therapy (MRgLITT) are increasingly being considered as an alternative to traditional open resections (e.g., formal anterior temporal lobectomy), in carefully selected patients with localized epileptogenic substrates like mesial temporal sclerosis. By delivering stereotactic thermal cytoreduction directly to epileptogenic hubs, MRgLITT significantly reduces cognitive morbidity and hospital length-of-stay in certain patients [152,153].

Nevertheless, both AI-guided closed-loop neuromodulation and ablative surgeries remain compensatory management strategies. While interrupting nodal propagation or ablating epileptogenic rich-club hubs effectively suppresses active ictogenesis and can substantially improve patients’ quality of life, these procedures do not restore physiological network homeostasis or reverse chronic neuroinflammatory and structural remodeling, underscoring once more the ongoing necessity for etiology-targeted disease modification. Also, long-term longitudinal data suggest that while these interventions can markedly reduce seizure burden, there is limited evidence that they halt the progression of cognitive comorbidities [5,109,116,154].

### 6.4. Anti-Inflammatory Agents and Organoid-Guided Interventions

Anti-inflammatory and immunomodulatory therapies represent one of the most promising frontiers for authentic disease-modifying interventions in temporal lobe epilepsy [14,67,123]. By targeting the microglial–cytokine axis and the HMGB1/TLR4 signaling cascade, small molecules aim to interrupt the chronic inflammatory loop that fuels ictogenesis and network degradation [72,73,75]. Selective antagonists such as Anakinra (a recombinant human IL-1 receptor antagonist) and VX-765 (a selective caspase-1 inhibitor that blocks IL-1β maturation) have demonstrated some preclinical efficacy in mitigating IL-1β-driven hyperexcitability, improving blood–brain barrier integrity, and raising seizure thresholds [122,123]. However, robust randomized clinical trials in chronic TLE are lacking. Concurrently, phytocannabinoids like cannabidiol (CBD) have evolved beyond pure symptomatic antiseizure administration; accumulating experimental evidence demonstrates that CBD exerts multi-target anti-inflammatory, antioxidant, and mitochondrial-stabilizing effects by suppressing reactive microglial activation and downregulating the NLRP3 inflammasome axis [75,155,156].

To better evaluate these targeted interventions and partially overcome species-specific translational barriers, the field is increasingly using human induced pluripotent stem cell (iPSC)-derived 3D cerebral organoids and assembloids. These can partially reproduce key cellular and network features, including aspects of cytoarchitecture, glial–neuronal signaling, and spontaneous network synchronization. These models provide complementary platforms for prioritizing candidate antiepileptogenic molecules and identifying potential off-target liabilities, while patient-specific organoids remain primarily a research tool rather than a routine clinical approach [157,158]. Unfortunately, the translation of these therapies into routine clinical management remains largely experimental. While biologics like Anakinra have shown promising efficacy in epilepsy associated with acute inflammatory encephalopathies (such as FIRES/NORSE) [159,160], their efficacy, optimal therapeutic window, and safety in chronic, established TLE are still under active investigation in preclinical studies and case-based clinical investigations [161]. Consequently, caution is warranted until large-scale, prospective clinical trials definitively establish the disease-modifying capacity and long-term safety of anti-inflammatory and biomarker- and human-model-informed regimens in human TLE [67,123].

## 7. Conclusions

The evolving landscape of epilepsy research underscores an ongoing paradigm shift, reframing temporal lobe epilepsy from a predominantly focal hippocampal seizure disorder into a dynamic, progressive, multiscale network disease. As outlined throughout this review, delineating the intricate molecular architecture of epileptogenesis provides a foundational blueprint for true disease modification. Grounding therapeutic strategies in this deep pathophysiological understanding enables a shift away from largely semiology- and syndrome-guided antiseizure medication treatment and from predominantly empirical, trial-and-error drug discovery toward etiology-driven precision medicine.

Moving forward, the integration of multimodal computational network connectomics, high-resolution rational drug design, targeted RNA therapeutics, and human iPSC-derived cerebral organoid platforms holds considerable promise for transforming patient outcomes in epilepsy. These approaches aim to target specific molecular and network mechanisms underlying epileptogenesis and seizure generation, rather than providing only symptomatic seizure control. However, translational expectations must be balanced with scientific realism: the majority of these mechanism-based, disease-modifying modalities remain highly experimental and are currently confined to preclinical models or early translational pipelines. Bridging the gap between laboratory models and clinical utility will require time, rigorous pharmacokinetic optimization, and extensive prospective human trials to establish long-term safety, CNS selectivity, and clinical efficacy. Importantly, while these novel therapies may address specific underlying mechanisms of epilepsy and epileptogenesis, much remains to be discovered if we are to meaningfully impact an increasing proportion of patients with refractory disease.

At this point, several key questions remain unresolved, among which the following are particularly important:

1. Can reliable, early-stage biomarkers identify epileptogenesis before recurrent seizures and irreversible network remodeling become established?
2. Can mechanism-based interventions selectively normalize pathological circuits without disrupting physiological network activity, development, cognition, or adaptive plasticity?
3. How can preclinical findings be translated across the biological and clinical heterogeneity of human TLE, including differences in etiology, age, disease stage, comorbidities, and treatment history?
4. Which combinations of biomarkers, patient-derived models, and longitudinal clinical endpoints can demonstrate genuine disease modification rather than transient seizure suppression?

Despite these unresolved questions, directly targeting the fundamental drivers of epileptogenesis rather than merely suppressing resultant ictogenesis remains a plausible long-term path toward preventing or ameliorating temporal lobe epilepsy and, ultimately, achieving disease modification.

## Data Availability

No new data were created or analyzed in this study. Data sharing is not applicable to this article.

## Abbreviations

The following abbreviations are used in this manuscript:

AI	Artificial intelligence
AMPA	α-Amino-3-hydroxy-5-methyl-4-isoxazolepropionic acid
AQP4	Aquaporin-4
ASM(s)	Antiseizure medication(s)
ASO(s)	Antisense oligonucleotide(s)
ATP	Adenosine triphosphate
BBB	Blood–brain barrier
BDNF	Brain-derived neurotrophic factor
CaBP(s)	Calcium-binding protein(s)
CA1	Cornu Ammonis area 1
CA4	Cornu Ammonis area 4
CBD	Cannabidiol
CB1	Cannabinoid receptor type 1
CB2	Cannabinoid receptor type 2
CCK	Cholecystokinin
CNS	Central nervous system
CR	Calretinin
CSF	Cerebrospinal fluid
DAMP(s)	Damage-associated molecular pattern(s)
DTI	Diffusion tensor imaging
EAAT1	Excitatory amino acid transporter 1
EAAT2	Excitatory amino acid transporter 2
EEG	Electroencephalography
EF-hand	Calcium-binding structural motif
FDG-PET	[^18F^]Fluorodeoxyglucose positron emission tomography
fMRI	Functional magnetic resonance imaging
GABA	γ-Aminobutyric acid
GABA_A_	Gamma-aminobutyric acid type A receptor
GFAP	Glial fibrillary acidic protein
GLT-1	Glutamate transporter 1
HARNESS-MRI	Harmonized Neuroimaging of Epilepsy Structural Sequences magnetic resonance imaging protocol
HFO(s)	High-frequency oscillation(s)
HMGB1	High-mobility group box 1
iNOS	Inducible nitric oxide synthase
iPSC	Induced pluripotent stem cell
KCC2	Potassium–chloride cotransporter 2
Kir4.1	Inwardly rectifying potassium channel 4.1
LPS	Lipopolysaccharide
MEG	Magnetoencephalography
miRNA(s)	MicroRNA(s)
MMP-9	Matrix metalloproteinase-9
MRgLITT	Magnetic resonance-guided laser interstitial thermal therapy
MRI	Magnetic resonance imaging
MTS	Mesial temporal sclerosis
mTOR	Mechanistic target of rapamycin
Na_v_	Voltage-gated sodium channel
Na^+^/K^+^-ATPase	Sodium–potassium adenosine triphosphatase
NECAB1	Neuronal calcium-binding protein 1
NLRP3	NOD-like receptor protein 3
NMDA	N-Methyl-D-aspartate
NKCC1	Sodium–potassium–chloride cotransporter 1
NORSE	New-onset refractory status epilepticus
NPY	Neuropeptide Y
P2X7	P2X purinoceptor 7
PAM(s)	Positive allosteric modulator(s)
PDS	Paroxysmal depolarization shift
PPARγ	Peroxisome proliferator-activated receptor gamma
PV	Parvalbumin
RAGE	Receptor for advanced glycation end-products
ROS	Reactive oxygen species
SCN1A	Sodium voltage-gated channel alpha subunit 1
SCN2A	Sodium voltage-gated channel alpha subunit 2
sEEG	Stereo-electroencephalography
SPECT	Single-photon emission computed tomography
STK-001	A targeted antisense oligonucleotide therapy for SCN1A-related Dravet syndrome
SUDEP	Sudden unexpected death in epilepsy
SV2A	Synaptic vesicle glycoprotein 2A
TANGO	Targeted Augmentation of Nuclear Gene Output
TGF-β	Transforming growth factor beta
TLE	Temporal lobe epilepsy
TLR4	Toll-like receptor 4
TNF-α	Tumor necrosis factor alpha
TrkB	Tropomyosin receptor kinase B
UBE3A	Ubiquitin protein ligase E3A
UBE3A-ATS	UBE3A antisense transcript
VX-765	A selective caspase-1 inhibitor
Y_2_	Neuropeptide Y receptor Y2
